# Neutrophil-lymphocyte ratio as a predictive biomarker for response to high dose interleukin-2 in patients with renal cell carcinoma

**DOI:** 10.1186/s12894-016-0192-0

**Published:** 2017-01-05

**Authors:** James A. Kuzman, David D. Stenehjem, Joseph Merriman, Archana M. Agarwal, Shiven B. Patel, Andrew W. Hahn, Anitha Alex, Dan Albertson, David M. Gill, Neeraj Agarwal

**Affiliations:** 1 University of Utah Huntsman Cancer Institute, Salt Lake City, UT USA; 2 Department of Pharmacotherapy, College of Pharmacy, University of Utah, Salt Lake City, UT USA; 3 Department of Pathology and ARUP Laboratories, University of Utah, Salt Lake City, UT USA

**Keywords:** Renal cell carcinoma, Neutrophil lymphocyte ratio, High dose interleukin-2

## Abstract

**Background:**

Immunotherapy with high-dose interleukin-2 (HD-IL2) results in long-term survival in some metastatic renal cell carcinoma (mRCC) patients but has significant acute toxicities. Biomarkers predicting response to therapy are needed to better select patients most likely to benefit. NLR (absolute neutrophil count (ANC)/absolute lymphocyte count (ALC)) is a prognostic and predicative biomarker in various malignancies. The goal was to determine whether NLR can predict response to HD-IL2 in this setting.

**Methods:**

Patients with clear cell mRCC treated with HD-IL2 were identified from an institutional database from 2003–2012. Baseline variables for the assessment of IMDC risk criteria, and neutrophil and lymphocyte count, were collected. Best response criteria were based on RECIST 1.0. Wilcoxon rank-sum test was used to evaluate the association of continuous baseline variables with disease control. NLR was stratified by ≤4 or >4. Progression free survival (PFS) and overall survival (OS) were estimated with the Kaplan-Meier method and Cox proportional hazard models assessed associations of NLR with survival.

**Results:**

In 71 eligible patients, median NLR in those with an objective response (*n* = 14, 20%) was 2.3 vs 3.4 in those without (*n* = 57, 80%, *p* = 0.02). NLR ≤4 was associated with improved progression free and overall survival. After adjustment for IMDC risk criteria, NLR remained a significant predictor of OS (ANC/ALC ≤4 vs >4, HR 0.41, 95% CI 1.09-5.46, *p* = 0.03; ANC/ALC continuous variable per unit change in NLR, HR 1.08, 95% CI 1.01-1.14, *p* = 0.03).

**Conclusions:**

In this discovery set, NLR predicts overall survival in patients treated with HD-IL2 in mRCC, and may allow better patient selection in this setting. Data needs validation in an independent cohort.

## Background

The landscape of metastatic renal cell carcinoma (mRCC) has significantly improved in the last decade as the biology is better understood and novel treatments are developed. Targeting various signal transduction molecules has been shown to be effective in improving progression free and overall survival. Despite these developments, the prognosis remains relatively poor, and most die of their disease within a few years of onset of metastatic disease. High dose interleukin-2 (HD-IL2) is an approved therapy for select patients with mRCC. It was one of the first immunotherapy agents used that resulted in a durable response in a small population of patients. However, the therapy is associated with many acute and rare chronic toxicities and requires experienced management of these acute toxicities in a critical care setting. Clearly a subset of patients derives benefit from HD-IL2, but at the current time there are no predicative markers to help identify these patients.

Prognostic models have been used for about a decade to help stratify patients with mRCC into different risk categories [1, 2]. However, currently there is not a single biomarker, which is used in the clinic to predict response to therapy in patients with mRCC. Current prognostic models include interval from diagnosis to treatment, Karnofsky performance status, serum LDH, corrected serum calcium, and serum hemoglobin. Later absolute neutrophil count greater than upper limit of normal was found to be an independent adverse prognostic factor [2]. Recently addition of NLR has been proposed to be used to help risk stratify patients with metastatic prostate cancer [3].

Neutrophil-lymphocyte ratio has been shown to be a prognostic marker for a wide variety of malignancies including renal cell carcinoma. It was also previously shown that increase in absolute lymphocyte number correlated with objective response in patients undergoing therapy with interleukin-2. Given that NLR is a crude measure of immune function it may be useful in predicting response with immune related treatments such as checkpoint inhibition or HD-IL2.

This study investigates the role of using NLR as a predicative marker of response to HD-IL2 in patients with mRCC. We hypothesized that lower NLR would be associated with better OR, PFS, and OS in patients treated with HD-IL2.

## Methods

### Study cohort

All sequential patients with clear cell mRCC treated with HD-IL2 at the University of Utah Huntsman Cancer Institute from 2003–2013 were identified. Any patient with clear cell mRCC with good performance status, and intact organ function was offered treatment with HDIL-2, regardless of the prognostic risk category. These are also the selection criteria currently recommended by the National Comprehensive Cancer Network (NCCN) guidelines for treatment with HDIL-2 [4]. Patients were excluded if date of last follow-up or death was not available or date of HD-IL2 administration was not recorded. Patient age, gender, Karnofsky performance status, and absolute neutrophil and lymphocyte values were collected prior to HD-IL2 therapy. Clear cell histology was confirmed by pathology reports and number and sites of metastasis prior to HD-IL2 was recorded. Demographics, as well as clinical and laboratory were collected. The Institutional Review Board of the University of Utah approved the study design, and informed consent was obtained from all patients.

### HD-IL2 treatment protocol

One course of HD-IL2 consisted of two cycles – cycle administered over 5–6 days, followed by one week off, followed by cycle two over 5–6 days. HD-IL2 dosing comprised the standard regimen of 600,000 IU/kg IV every 8 h for a total of 14 planned doses per cycle. Restaging scans were done approximately 8 weeks after the first course. Thereafter, restaging scans were done every 12 weeks.

### HD-IL2 response criteria

Best response criteria were based on RECIST 1.0. A PR was defined as a >30% decrease in target lesion size. Progressive disease was a >20% increase in target lesion size or new lesion. CR indicated no imaging evidence of disease. Patients not meeting criteria for PR or progressive disease (PD) were considered to have stable disease (SD). Patients without appropriate follow-up between radiographic imaging and treatment, who were lost to follow-up or died before determining response were classified as not evaluable (NE) and grouped with PD for statistical analysis.

### NLR ratio

The absolute neutrophil and lymphocyte values immediately prior to initiating HD-IL2 and within 30 days were used to calculate the NLR ratio. The 75% quartile of NLR values was used to stratify outcomes. The NLR was also assessed as a continuous variable.

### Objective

The primary objective was progression free survival (PFS) and overall survival (OS) stratified by NLR patients with mRCC treated with HD-IL2.

### Statistical analysis

Descriptive statistics were used to summarize patient and treatment characteristics. Kaplan-Meier method with log-rank tests were used to assess PFS and OS by HD-IL2 response. PFS was defined as the time from first HD-IL2 initiation to disease progression, death, or last follow-up. OS was defined as the time from first HD-IL2 administration to death or last follow-up. In the PFS analysis, censoring occurred at the time of treatment discontinuation if treatment was discontinued for any other reason than progression or death. In both the PFS and OS analysis, censoring occurred at the time of last follow-up in those who had not progressed or were still alive at the end of the designated study period. Cox proportional-hazards models were created with IMDC prognostic risk criteria, gender, and NLR ratio both as a continuous variable and with a cut off of at the 75% quartile for PFS and OS. Significance was set at less than 0.05 for the analysis.

## Results

**Fig. 1:**
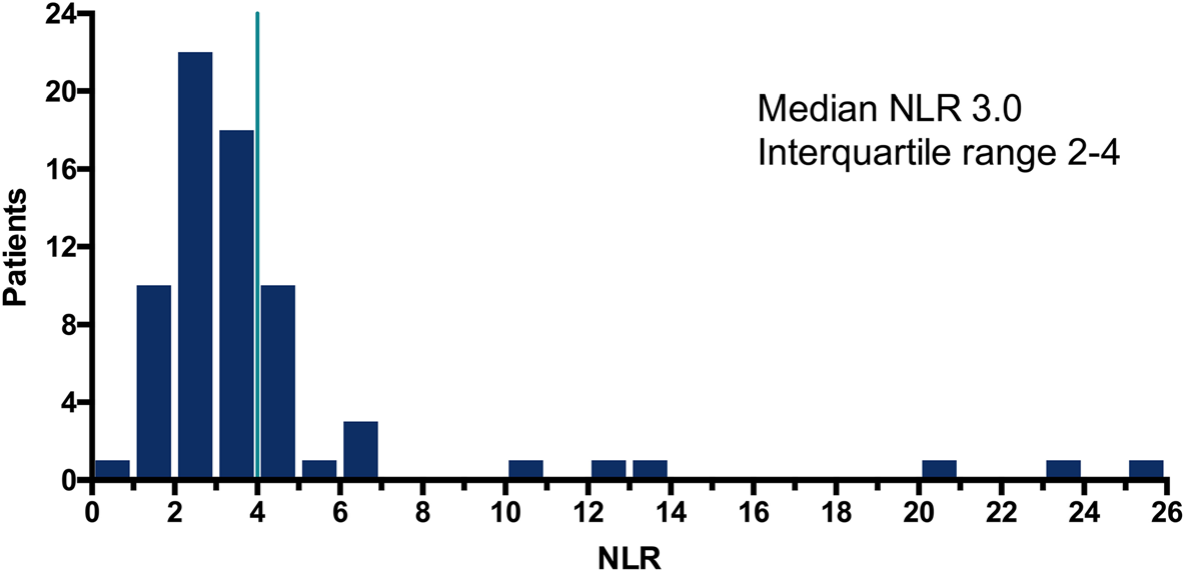
Histogram of the NLR at initiation of HD-IL2

**Fig. 2:**
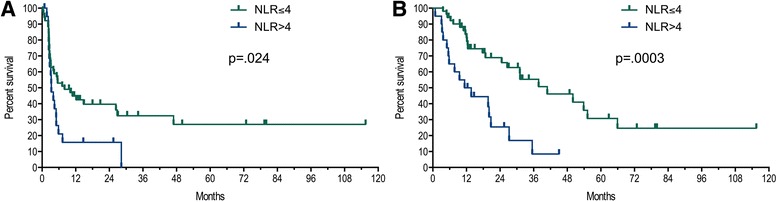
Progression-Free survival (**a**) and Overall Survival (**b**) stratified by NLR ≤4 vs >4

In 71 eligible patients ANC and ALC values were obtained and 53 (75%) of the patients were male with a median age at diagnosis of metastatic disease of 55 years. IMDC criteria was favorable for 9 (13%), intermediate for 49 (69%), and poor for 13 (18%) patients (Table 1). The median NLR was three and the 75% quartile was 4 (Fig. 1). There was a trend for better objective response rate in patients with NLR < 4 though this was not significant (24% vs 10%, *p* = 0.32). There was also a trend for higher complete response rate in patients with NLR <4 vs ≥4 with CR rates of 18% vs 0% (*p* = 0.086), respectively (Tables 1 and 2). NLR ≤4 (versus NLR >4) was associated with significant improvement in both progression free and overall survival (Figs. 2a and 1b). Median PFS was improved by 4.7 months (8.0 vs. 3.3, *p* = 0.024), and median OS was improved by 28.4 months (40.9 vs 12.5, *p* = 0.0003). The role of NLR to predict survival outcomes after adjustment for IMDC risk criteria was also investigated. NLR was significant predictor of PFS in univariate analysis and OS by univariate and OS for multivariate analysis after correction for IMDC criteria and sex (Table 3).

**Table 1:** Demographics and disease characteristics between NLR ≤4 vs NLR >

	NLR ≤ 4 *n* = 51	NLR > 4 *n* = 20	*P*-value
Age			
Years of age, median (IQR)	55 (49–58)	55 (49–59)	0.9^a^
Sex			
Males, n (%)	42 (82%)	11 (55%)	*0.021* ^b^
Prior Therapy, n (%)			
Nephrectomy	51 (100%)	20 (100%)	-
Previous systemic treatment	7 (14%)	6 (30%)	0.12^b^
Number of metastatic disease sites, n (%)			
1	11 (22%)	6 (30%)	0.40^c^
2	15 (29%)	2 (40%)	
3	12 (24%)	5 (25%)	
≥ 4	13 (26%)	7 (35%)	
IMDC risk factors, n (%)			
Favorable	7 (14%)	2 (10%)	*0.013* ^c^
Intermediate	39 (76%)	10 (50%)	
Poor	5 (10%)	8 (40%)	

^a^Wilcoxon Rank Sum

^b^Chi-Square

^c^Fisher’s Exact

Italicized values are less than 0.05

**Table 2:** Best Reponses to HD-IL2 between NLR ≤4 vs NLR >4

	NLR ≤ 4 *n* = 51	NLR > 4 *n* = 20	*P*-value
Best response			
CR	9 (18%)	0 (0%)	0.086^a^
PR	3 (6%)	2 (10%)	
SD	18 (35%)	5 (25%)	
PD/NE	21 (41%)	13 (65%)	
Objective response	12 (24%)	2 (10%)	0.32^a^
Clinical benefit	30 (59%)	7 (35%)	0.11^b^

^a^Fisher’s Exact

^b^Chi-Square

**Table 3:** Univariate and Multivariate analysis results for PFS and OS

Variable	Univariate		Multivariate Model 1		Multivariate Model 2	
	PFS HR (95% CI), *P*-value	OS HR (95% CI), *P*-value	PFS HR (95% CI), *P*-value	OS HR (95% CI), *P*-value	PFS HR (95% CI), *P*-value	OS HR (95% CI), *P*-value
Sex						
Male vs Female	0.49 (0.27-0.93), *p = .031*	0.35 (0.17-0.71), *p = .005*	0.64 (0.33-1.28), *p* = .20	0.67 (1.09-5.46), *p* = .33	0.56 (0.30-1.08), *p* = .08	0.45 (0.22-0.93), *p = .03*
IMDC Criteria						
Favorable	ref	ref	ref	ref	ref	ref
Intermediate	2.20 (0.93-6.50), *p* = .08	4.13 (1.23-25.62), *p = .02*	2.02 (0.83-6.06), *p* = .13	4.02 (1.16-25.33), *p = .03*	1.91 (0.78-5.73), *p* = .17	3.42 (1.00-21.48), *p = 0.05*
Poor	4.46 (1.56-14.65), *p = .005*	10.43 (2.65-69.34), *p = .0004*	3.38 (1.12-11.55), *p = .03*	7.00 (1.68-47.83), *p = .006*	3.23 (1.03-11.30), *p = .04*	5.41 (1.20-38.24), *p = .03*
NLR						
≤ 4 vs >4	0.51 (0.28-0.95), *p = .034*	0.31 (0.16-0.61), *p = .001*	0.68 (0.35-1.39), *p* = .28	0.41 (1.09-5.46), *p = .03*	NA	NA
Continuous (per unit change in NLR	1.05 (0.99-1.10), *p* = .08	1.10 (1.04-1.15), *p = .002*	NA	NA	1.03 (0.97-1.09), *p* = .30	1.08 (1.01-1.14), *p = .03*

CI, 95% confidence interval

*HR* hazard ratio, *NA* not applicable, *NLR* neutrophil to lymphocyte ratio, *OS* overall survival, *PFS* progression-free survival

Italicized *p*-values are less than 0.05

## Discussion

This study shows that NLR could be used to help predict response to HD-IL2. HD-IL2 is a very effective treatment for a small population of patients with mRCC. Given its several acute but rare chronic toxicities, a predictive biomarker in this setting is expected to optimize selection of patients, who are most likely to derive benefit from therapy. Low NLR has been associated with a better prognosis for many different types of malignancies. This is the first report to suggest that low NLR may be “predictive” of improved survival outcomes to HD-IL2 in the setting of mRCC.

It is clear that inflammation and immune response play a pivotal role in neoplastic progression [5]. Indeed novel treatment strategies targeting the immune system, such as immune check point inhibitors, have been shown to improve outcomes and are approved for multiple malignancies. One of simplest estimations of the balance of inflammation and immune response is neutrophil/lymphocyte ratio [6, 7].

NLR has been reported to be a predictive and prognostic factor for localized renal cell carcinoma [8, 9]. In a large meta-analysis of 15 cohorts including 3357 patients, NLR predicted poorer OS (hazard ratio = 1.82, 95% CI 1.51-2.19) [10]. Additionally, high preoperative NLR was associated with larger tumor size, higher nuclear grade, histologic tumor necrosis, and sarcomatoid differentiation [8]. Recently, on treatment neutropenia was shown to be an independent biomarker of favorable outcome in mRCC, independent of treatment type [11]. NLR was also recently shown to predict response to ipilimimab in melanoma patients. In a recent report, lower NLR ratio predicted improved overall survival in patients with metastatic melanoma [12].

Unlike recently developed immunotherapeutic agents, the mechanism of action of HD-IL2 is not fully understood. Interleukin-2 is a recombinant protein that has a wide range of effects on the immune system, including promoting proliferation and differentiation of CD4(+) T cell into specific effector T cell subsets, of CD8(+) T cells into effector T cells, and in to memory cells, but also expansion of immunosuppressive CD4(+)FOXP3 T regulatory cells in certain situations [13].

Historically, HD-IL2 therapy has generally been shown to have an objective response rate of approximately 10-20%, including complete responses in ~10% of patients. More recently, in a large cohort of patients with mRCC (*n* = 391) treated with HDIL-2, a clinical benefit with HD-IL2 was seen in ~50% of patients. In addition to ~20% patients who experienced objective responses (CR in 9% and PR in 10%), an additional 32% experienced SD as the best response to treatment. The survival outcomes were similar in those experiencing PR and SD, and were significantly superior to those who did not experience objective responses or SD [14]. Although the use of HDIL-2 declined after the approval of targeted therapies starting in 2005, in the recent years with the resurgence of cancer immunotherapy in general, the use of HDIL-2 has stabilized and may have picked up [15]. Identification of predictive biomarkers in this setting is expected to further allow more patients to experience benefits of HDIL-2 while limiting toxicities and cost in others. No other therapy in the mRCC setting has been shown to be associated with durable long-term response, albeit in a small proportion of patients, in a consistently reproducible fashion. Absolute number of peripheral blood lymphocytes have been correlated with objective response in patients treated with IL-2, interferon alpha, and histamine. There was no difference in baseline levels of lymphocytes of responding versus non-responding patients [16]. This further supports that NLR probably acts as a better marker to predict response in patients with mRCC treated with HD-IL2.

One of the main limitations of this study is the retrospective nature of the study and the relatively small sample size.

## Conclusion

In conclusion, these hypotheses generating data provides initial evidence that low NLR may predict improved survival outcomes in mRCC, and help better selection of patients for HD-IL2 therapy. Low NLR was associated with significantly improved PFS and OS with a trend for improved objective responses with HD-IL2. Data need further validation in a larger and an independent cohort.

## Abbreviations

ALC: Absolute lymphocyte count
ANC: Absolute neutrophil count
CI: Confidence interval
HD-IL2: High-dose interleukin-2
HR: Hazard ratio
mRCC: Metastatic renal cell carcinoma
NE: No effect
NLR: Neutrophil to lymphocyte ratio
OS: Overall survival
PD: Progressive disease
PFS: Progression free survival
SD: Stable disease
